# Usefulness of dynamic contrast-enhanced MRI in the evaluation of osteonecrosis of the proximal fragment in scaphoid fractures

**DOI:** 10.1590/0100-3984.2017.0036

**Published:** 2018

**Authors:** Luiza Werneck, Clarissa Canella, Flavia Costa, Alessandro Severo Alves de Melo, Edson Marchiori

**Affiliations:** 1 Clínica de Diagnóstico Por Imagem (CDPI), Rio de Janeiro, RJ, Brazil.; 2 Clínica de Diagnóstico Por Imagem (CDPI), Rio de Janeiro, RJ, e Universidade Federal Fluminense (UFF), Niterói, RJ, Brazil.; 3 Universidade Federal Fluminense (UFF), Niterói, RJ, Brazil.; 4 Universidade Federal do Rio de Janeiro (UFRJ), Rio de Janeiro, RJ, Brazil.

*Dear Editor,*

**Figure 1:**
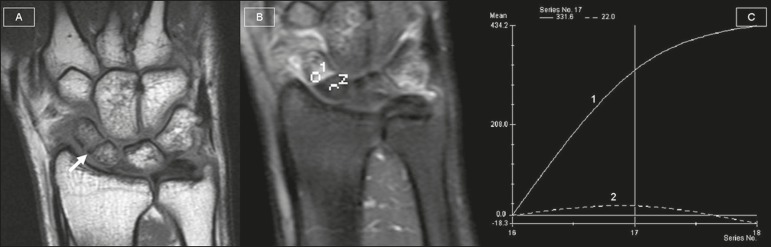
Coronal T1-weighted image showing a fracture line through the scaphoid waist (A). Dynamic gadolinium contrast-enhanced coronal T1-weighted image with fat saturation showing the complete absence of enhancement of the proximal pole of the scaphoid (B), as well as a time-signal intensity curve (C) lower than that of the distal pole, with a maximum enhancement of 50%, denoting satisfactory perfusion of the proximal pole.

A 26-year-old man who had fractured his scaphoid four weeks previously presented with persistent wrist pain. Magnetic resonance imaging (MRI) showed a fracture line through the scaphoid waist (Figure 1). A gadolinium contrast-enhanced coronal T1-weighted sequence with fat saturation was acquired, as were time-signal intensity curves of the proximal and distal scaphoid poles. The complete absence of enhancement of the proximal pole of the scaphoid, together with the fact that the time-signal intensity curve was lower for the proximal pole than for the distal pole, denoted satisfactory perfusion of the proximal pole.

The scaphoid is the most commonly fractured bone of the carpus, and healing is interrupted by nonunion in 5-15% of cases. Scaphoid fracture nonunion may progress to avascular necrosis of the scaphoid in cases of long-standing nonunion, after failed surgery, in certain fractures of the proximal third of the scaphoid, or when an occult fracture is not treated. The proximal pole of the scaphoid is prone to avascular necrosis due to the distal location of the main feeding vessels and the retrograde pattern of the intraosseous blood supply. Stress fracture of the scaphoid waist is believed to contribute to osteonecrosis of the scaphoid resulting from repetitive dorsiflexion of the wrist, the waist being the weakest point in the scaphoid^(1-4)^.

A number of recent studies conducted in Brazil have highlighted the importance of MRI in the evaluation of diseases affecting the musculoskeletal system^(5-9)^. The use of a reliable noninvasive diagnostic tool to assess the viability of the proximal scaphoid pole is necessary to help surgeons plan the treatment of scaphoid nonunion, because there is currently no consensus regarding when conservative or surgical treatment is indicated. Recently, gadolinium contrast-enhanced MRI has been shown to be the most accurate modality for evaluating scaphoid viability. In fact, some authors have suggested that dynamic contrast-enhanced MRI represents a valuable tool in assessing whether conservative or surgical treatment is indicated to achieve a good functional outcome ^(1,4)^. According to those authors, if dynamic contrast-enhanced MRI shows poor perfusion of the proximal pole of the scaphoid, primary surgical intervention would be indicated. Despite the fact that time-signal intensity curves of the proximal and distal scaphoid poles are actually widely used, there have been a few reports suggesting that the analysis of these curves does not provide additional predictive value over standard delayed enhancement MRI for acute scaphoid fracture^(1-4)^.
